# Chromatin condensation dynamics during spermatogenesis and variation in detectability and spatial distribution of satellite DNAs outside constitutive heterochromatin in *Tenebrio molitor*

**DOI:** 10.3389/finsc.2026.1858332

**Published:** 2026-05-28

**Authors:** Patrik Majcen, Eva Šatović-Vukšić

**Affiliations:** Laboratory for Molecular Genetics, Ruđer Bošković Institute, Division of Molecular Biology, Zagreb, Croatia

**Keywords:** chromatin condensation, FISH, H3K9me3, heterochromatin, IF, satellite DNA, *Tenebrio molitor*

## Abstract

Repetitive DNA sequences comprise a significant proportion of eukaryotic genomes and play an important role in genome structure, function, and evolution. The genome of the beetle *Tenebrio molitor* is particularly rich in satellite DNAs (satDNAs), which represent a prominent component of its repetitive DNA fraction. While dominantly found in long arrays confined to heterochromatin, satDNAs can also be found in short arrays in extra-heterochromatic regions. However, their cytogenetic detectability and spatial distribution can be influenced by chromatin condensation and epigenetic context. In this brief report, we investigated chromatin condensation dynamics during male germline development in *T. molitor* and examined how these dynamics affect the detection and localization of high- and low-copy satDNAs. DNA staining, immunodetection of the H3K9me3 epigenetic mark of constitutive heterochromatin, and fluorescence *in situ* hybridization (FISH) of satDNA sequences were used to examine the relationship between chromatin organization, the functional state of heterochromatin, and satDNAs positioning and detectability. Our results revealed that a large, central, and highly condensed chromosomal core is enriched in H3K9me3 and corresponds to constitutive heterochromatin. Major satDNA forms the bulk of this compartment and remains readily detectable regardless of chromatin condensation level. In contrast, the detectability of low-copy satDNAs decreased with increasing chromatin condensation. Additionally, numerous weak, punctate signals belonging to low-copy satDNAs were observed outside the DAPI-bright chromosomal mass, located in very loosely condensed chromatin, largely excluded from the heterochromatic chromosomal core. These findings demonstrate that chromatin condensation and constitutive heterochromatin organization strongly influence satDNA detectability and spatial distribution in *T. molitor*.

## 1 Introduction

The yellow mealworm, *Tenebrio molitor*, has increasingly gained attention as a model organism across a range of disciplines, including biology, biochemistry, immunology, physiology, evolutionary and repetitive DNA research (1–10). Owing to its high protein content and nutritional value, *T. molitor* is increasingly utilized as an animal feed supplement and has also been introduced into the human food market (11–13). In addition, this species produces antifreeze proteins with applications in vegetable preservation (14). Extracts from *T. molitor* have shown antiproliferative effects against colorectal adenocarcinoma and hepatocellular carcinoma (15), as well as antimicrobial and wound-healing activities (16, 17). The relevance of this species is further emphasized by ongoing efforts to improve its genome assembly and annotation (9, 18, 19), and the recent improved genome assembly was accompanied by satellitome analysis (9).

Chromatin organization undergoes profound and tightly regulated changes during male sperm cells development. In many eukaryotes, spermatogenesis is accompanied by progressive chromatin condensation, large-scale nuclear reorganization, and transitions between transcriptionally permissive and repressive chromatin states (20). These processes are essential for genome stabilization, regulation of gene expression, and the establishment of the highly compacted chromatin characteristic of differentiated male germ cells. Although chromatin dynamics during spermatogenesis have been extensively studied in different models (21), they remain insufficiently characterized in many beetle species, including *T. molitor*.

During spermatogenesis, early spermatogonia and spermatocyte stages are generally characterized by relatively decondensed chromatin that supports active transcription and meiotic recombination. As cells progress through meiosis and enter spermiogenesis, chromatin becomes progressively compacted, often accompanied by large-scale reorganization of nuclear architecture and the expansion of heterochromatic domains (21). A key epigenetic hallmark of constitutive heterochromatin is trimethylation of histone H3 at lysine 9 (H3K9me3), a conserved modification associated with transcriptional repression, repetitive DNA silencing, and maintenance of genome stability (22, 23). H3K9me3 is typically enriched at pericentromeric regions and repetitive DNA arrays, and plays a central role in heterochromatin establishment and propagation (24).

Satellite DNAs (satDNAs) are tandemly repeated sequences that frequently accumulate in heterochromatic regions of eukaryotic chromosomes. SatDNAs are now recognized as dynamic genomic elements with potential roles in chromosome segregation, nuclear architecture, and gene regulation [reviewed in (25, 26)]. Long satDNA arrays are commonly embedded within heterochromatin; however, not all satDNA sequences are confined to heterochromatic blocks. Increasing evidence suggests that low-copy or dispersed satDNA segments may occur outside major heterochromatic domains, occupying euchromatic or facultative heterochromatic regions [reviewed in (27)].

In Tenebrionidae, studies have documented prominent heterochromatic regions and abundant satDNAs (28, 29). In *T. molitor*, eleven satDNA families have been identified. The major 142-bp satDNA, designated TmSat01 (9), whose abundance estimations vary and amount up to 50% of the genome, is located in large pericentromeric heterochromatic blocks that extend along nearly the entire length of the chromosomes (10, 30–33). In addition to the major satDNA, ten additional satDNA families with substantially lower abundance have been identified (TmSat02–TmSat11), representing between 0.45% (TmSat02) and 0.01% (TmSat11) of the genome (9). The basic cytogenetic characterization of the chromosomal distribution of *T. molitor* satDNAs has been reported in (10); however, that study did not specifically address the differences in detection efficiency across different chromatin condensation stages or the specificities in the spatial distribution of satDNAs which are located outside heterochromatic domains.

*T. molitor* has a diploid chromosome number of 2n = 20, as determined by early cytogenetic studies (34). In this species, constitutive heterochromatin has been previously identified primarily using C-banding techniques (32, 33). While C-banding provides valuable information on the distribution of heterochromatin at the chromosomal level, it is based on differential staining properties and does not directly reflect the underlying epigenetic state. In contrast, immunodetection of H3K9me3 enables the visualization of heterochromatin based on a conserved molecular hallmark associated with transcriptional repression and stable heterochromatin formation (22, 23). Therefore, H3K9me3 immunofluorescence offers a more mechanistically informative approach, allowing assessment of heterochromatin organization while providing insight into its functional state.

In this study, we (i) report the dynamics of chromatin condensation during male germline development in *T. molitor*, (ii) report the trends of detectability of high- and low-abundance satDNAs depending on the degree of chromatin condensation, (iii) map constitutive heterochromatin domains based on the H3K9me3 epigenetic mark detection, and (iv) assess the specificities in the positioning of low-copy satDNA sequences found outside of constitutive heterochromatin.

## 2 Materials and methods

### 2.1 Chromosome spread preparation from male gonadal tissue

Pupal sex was determined according to the protocol described by Bhattacharya et al. (35). Chromosome spreads were prepared from male pupae. Testes were dissected and incubated in distilled water for 45 minutes to induce osmotic shock. The material was subsequently fixed in freshly prepared Carnoy’s fixative (absolute ethanol:glacial acetic acid, 3:1) and then dissociated in 50% glacial acetic acid in an Eppendorf tube. The resulting cell suspension was dropped onto clean glass slides and air-dried on a hot plate at 42°C. Once dried, the preparations were dehydrated through ethanol series graded 70%, 90%, and 100%, 30 s in each. For the detection of different stages of male gonadal cells development, slides were stained with DAPI (4′,6-diamidino-2-phenylindole) (Sigma-Aldrich) and mounted with Mowiol 4-88 (Sigma-Aldrich) mounting medium. Slide visualization was performed using a Zeiss Axio Observer fluorescent microscope. Stages of gonadal cells were determined according to (34). Cell types, division stages and ploidy transitions during spermatogenesis are listed in **Supplementary Table 1**.

### 2.2 H3K9me3 immunofluorescence

Slides were rinsed in 1× PBS for 2min, followed by two washes in 1× PBS supplemented with 0.2% Tween 20 (5 min each). Blocking was carried out in 2.5% BSA prepared in 1× PBS/0.2% Tween 20 for 1h at 37°C. The preparations were then incubated overnight at 37°C in a humid chamber with rabbit anti-H3K9me3 primary antibody (Cell Signaling) diluted 1:400 in 1× PBS containing 0.2% Tween 20 and 2.5% BSA. After primary antibody incubation, slides were washed three times in 1× PBS/0.2% Tween 20. The secondary antibody (donkey anti-Rabbit IgG Alexa Fluor 594, Invitrogen) was applied at a dilution of 1:1000 in 2.5% BSA in 1× PBS/0.2% Tween 20 and incubated for 1h at 37 °C in a humid chamber. Following that, slides were washed twice in 1× PBS/0.2% Tween 20 (5 min each) and once in 1× PBS for 2 min. Finally, preparations were counterstained with DAPI and mounted in Mowiol 4-88 (Sigma-Aldrich) mounting medium.

### 2.3 Fluorescence *in situ* hybridization

DNA probes for fluorescence *in situ* hybridization (FISH) were generated by PCR labeling. Primer sequences and PCR amplification conditions are listed in **Supplementary Table 2**. Each 20 µL reaction contained 20 ng of template DNA, 2.5 U GoTaq Flexi G2 DNA polymerase (Promega), GoTaq reaction buffer, 1.5 mM MgCl_2_, 0.1 µM of each primer, 100 µM dATP, dCTP, and dGTP (NEB), 50 µM dTTP (NEB), and 50 µM biotin-16-dUTP (Jena Bioscience). Following amplification, the products were verified by agarose gel electrophoresis and subsequently purified using either the QIAquick PCR Purification Kit or the QIAquick Gel Extraction Kit (Qiagen). Probe concentrations were quantified with a DeNovix fluorometer.

FISH analyses were conducted according to the protocol of (36), with a modified pepsin digestion step performed for 5 min at 37 °C. Each hybridization mixture contained 50 ng of satDNA probe. Probes were denatured at 80 °C for min and rapidly chilled on ice for 2min prior to application. Chromosomal DNA was denatured in 50% formamide (Sigma-Aldrich) prepared in 2× SSC for 1 min and 45 s. Hybridization signals were detected using fluorescein-conjugated streptavidin (Vector Laboratories) diluted 1:200, followed by biotinylated anti-streptavidin (Vector Laboratories) at a 1:100 dilution, and a second amplification step with fluorescein-conjugated streptavidin (1:200). Chromosomes were counterstained with DAPI (Sigma-Aldrich) and mounted in Mowiol 4-88 (Sigma-Aldrich). Fluorescent signals were examined and imaged using a Leica TCS SP8 X laser-scanning confocal microscope. In *T. molitor*, TmSat02 – TmSat11 represent low-copy satDNA families. Among these, TmSat09, TmSat10, and TmSat11 each produced a single signal on one chromosome pair (10). From the remaining low-copy satDNAs, which exhibited comparable trends of hybridization patterns across chromatin condensation states, TmSat02, TmSat04, TmSat06, and TmSat08 were selected for presentation as representatives. TmSat01, as the sole high-copy satDNA, was included as a reference for highly abundant repeats. Approximately 25 nuclei were analyzed for each satDNA probe.

## 3 Results

### 3.1 Chromatin condensation dynamics during spermatogenesis

**Figure 1:**
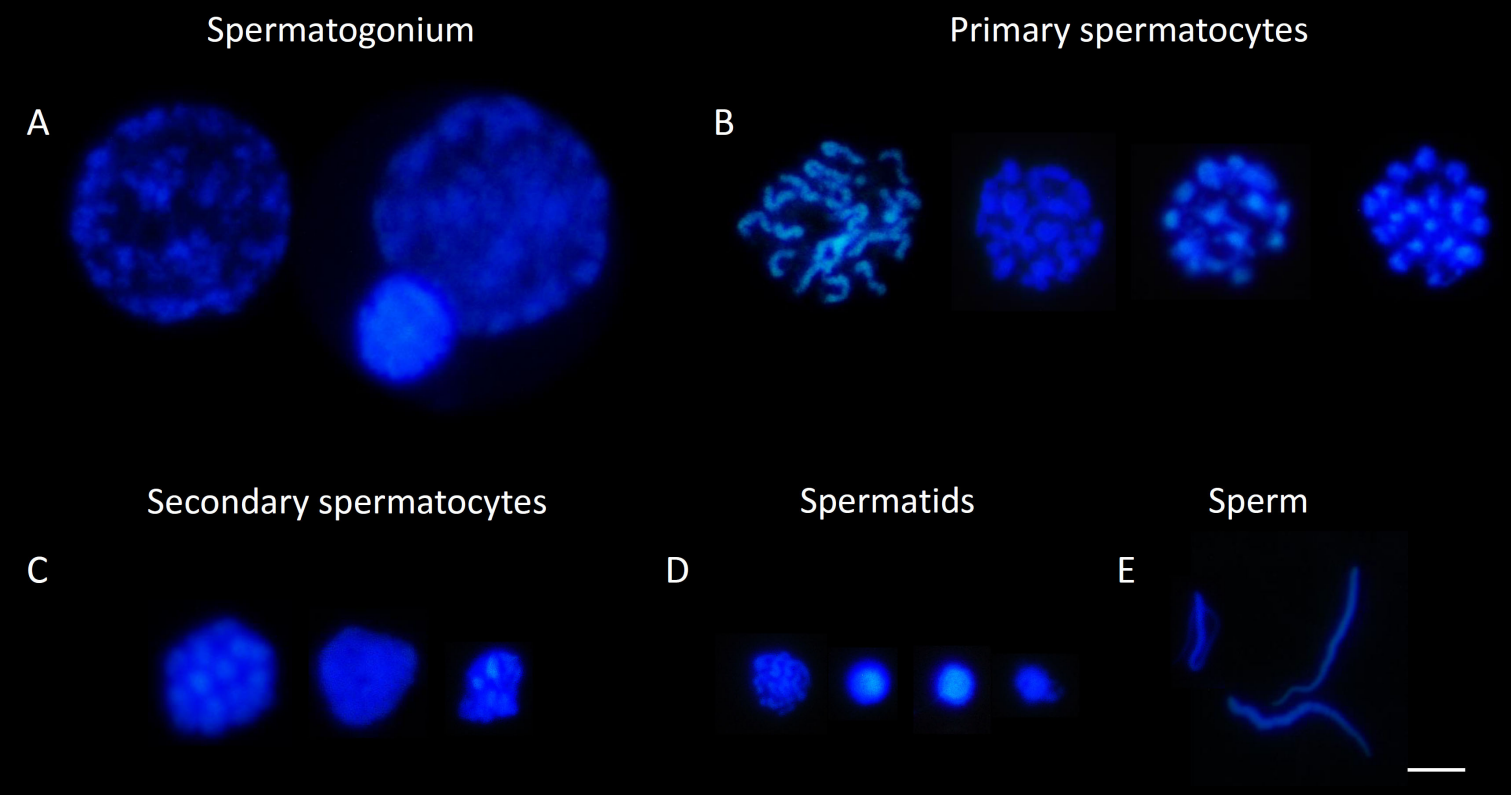
Male gonadal cells of *T. molitor*. DNA was stained with DAPI (blue). For each gonadal cell type, several chromatin condensation stages are presented. Spermatogonia **(A)**, primary spermatocytes **(B)**, secondary spermatocytes **(C)**, spermatids **(D)**, and mature spermatozoa **(E)**. Scale bar represents 5 µm.

We have inspected chromatin condensation dynamics across different stages of male gonadal cell development in *T. molitor* spermatogenesis. Spermatogonia are distinguished by a large nucleus with largely decondensed chromatin (**Figure 1A**). Primary spermatocytes undergoing meiotic prophase display various nuclear morphologies corresponding to differences in chromatin condensation at distinct substages (**Figure 1B**). Secondary spermatocytes are characterized by smaller, more compact nuclei containing extensive dense chromatin masses (**Figure 1C**). As cells transition into the spermatid stage, chromatin condensation intensifies and nuclear size decreases further, yielding small, round, and densely stained spermatids (**Figure 1D**). The fully mature spermatozoa acquire an elongated shape, accompanied by thinning along the tail region (**Figure 1E**).

### 3.2 Differential detection of high- and low-copy satDNAs in relation to chromatin condensation levels

**Figure 2:**
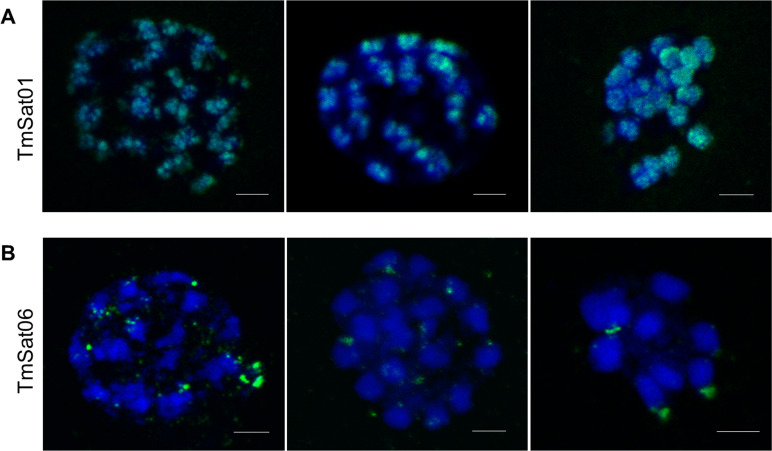
Fluorescence *in situ* hybridization localization of *T. molitor* TmSat01 and TmSat06 satDNAs across different chromosomal condensation stages of spermatocytes. Stages TmSat01 (left to right): pachytene/early diplotene, diplotene, and prometaphase I of primary spermatocytes. TmSat06 (left to right): pachytene/early diplotene of primary spermatocytes, diplotene of primary spermatocytes, and early metaphase II of secondary spermatocytes. SatDNA signals are shown in green, and chromosomes are counterstained with DAPI (blue). Scale bar represents 3 µm. **(A)** TmSat01, **(B)** TmSat06.

Differences in detectability were observed between high and low-abundant satDNA families of *T. molitor* depending on the level of chromatin condensation. High-copy satDNA TmSat01 produced strong and consistent hybridization signals and remained readily detectable regardless of chromatin condensation level (**Figure 2A** panels). In contrast, low-copy satDNA, exemplified by TmSat06, showed reduced detectability with increasing chromatin condensation. While numerous interspersed signals could be observed in less condensed chromatin regions, their number decreased upon chromatin condensation. This was particularly pronounced for weak signals derived from short interspersed arrays, with a number of signals becoming weak and ultimately not detectable (panels within **Figure 2B**).

### 3.3 Detection of *T. molitor* constitutive heterochromatin via H3K9me3 epigenetic mark

**Figure 3:**
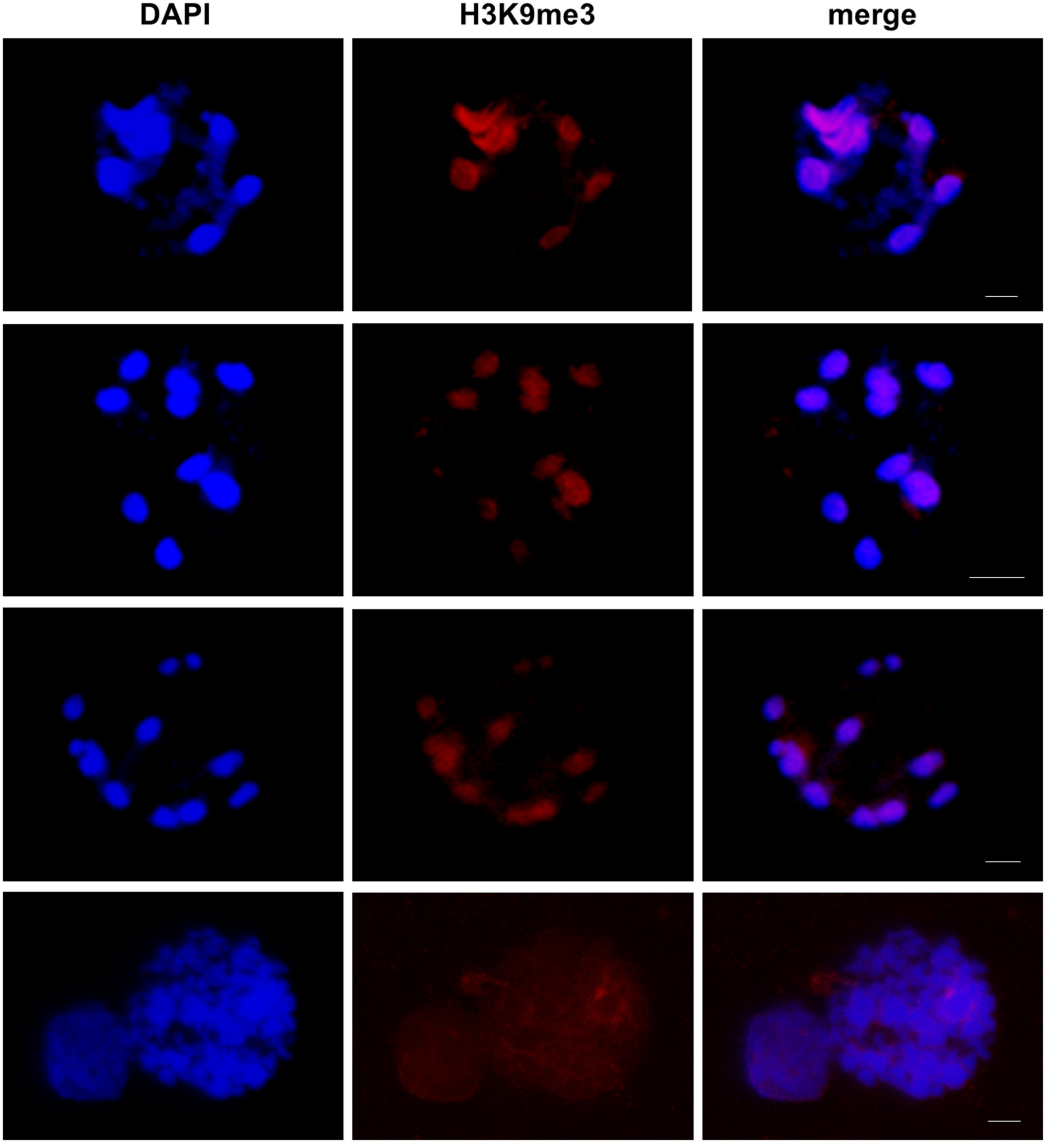
Immunofluorescent detection of the heterochromatin mark H3K9me3 in *T. molitor* nuclei. Rows show: (1) prometaphase of secondary spermatocytes; (2) metaphase chromosomes of secondary spermatocytes; (3) prometaphase of primary spermatocytes; and (4) spermatogonial DNA. Panels show DAPI staining (blue), H3K9me3 signal (red), and merged images. Scale bar represents 3 µm.

In order to address the localization and distribution of constitutive heterochromatin we have performed immunofluorescence with the antibody to H3K9me3 epigenetic mark. Immunostaining revealed that H3K9me3 signals occupy large parts of the chromosomal length on all *T. molitor* chromosomes, encompassing the majority of the chromosomal core (**Figure 3**, panels 1-3). In the decondensed chromatin state, these sequences can be observed as weak, less dense signals distributed over much of the nuclear DNA (**Figure 3**, last panel).

### 3.4 SatDNAs localization outside of the condensed chromosomal mass

**Figure 4:**
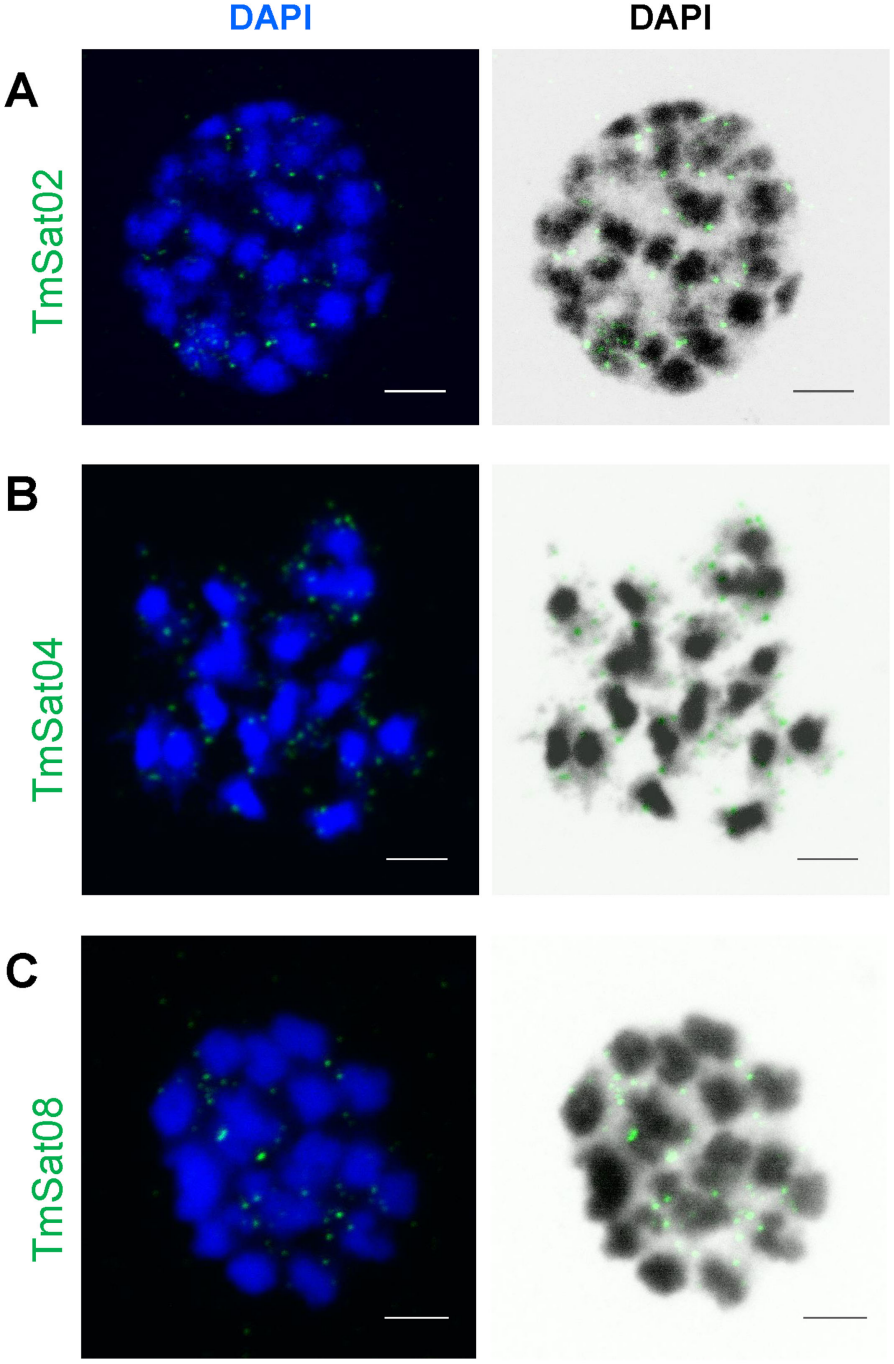
Peripheral distribution of low-copy satDNA signals in loosely condensed chromatin of primary spermatocytes in prophase I in *T. molitor*. Panels show merged images of satDNA FISH signals (green) with DAPI staining in blue together with the corresponding inverted DAPI images (black and white) to visualize loose chromatin distribution and satDNA positioning. Scale bar represents 3 µm. **(A)** TmSat02, **(B)** TmSat04, **(C)** TmSat08.

FISH hybridization revealed that several low-copy satDNAs of *T. molitor* generate numerous punctate signals, strongly localized to peripheral chromosomal regions. These signals are visible as scattered dots surrounding the more intensely DAPI-stained chromosomal core, and initially appeared to lie outside the DAPI-stained chromatin (**Figure 4**). However, visualization of the inverted DAPI image revealed that less condensed chromatin forms a halo-like area surrounding the central, more strongly condensed chromosomal mass, characterized by intense DAPI staining. Within this peripheral region, satDNA signals consistently corresponded to regions of very loosely condensed chromatin (**Figure 4**).

## 4 Discussion

Our cytogenetic analyses provide a detailed view of chromatin condensation dynamics throughout male germline development in *T. molitor*. Chromatin undergoes a progressive transition from a relatively open, toward a highly compacted configuration as spermatogenesis proceeds (**Figure 1**), which is consistent with observations in other species, e.g. *Drosophila* (21). In *T. molitor* we observed that nuclei retain regions of less condensed chromatin that contrast sharply with a central core of densely stained chromosomal mass, as revealed by DAPI staining and inverted-DAPI imaging (**Figure 4**). This architecture indicates the heterochromatin–euchromatin organization where constitutive heterochromatin coalesces into a central core while more loosely packed regions extend peripherally. We mapped constitutive heterochromatin domains in *T. molitor* using H3K9me3, a hallmark of transcriptionally silent, tightly condensed chromatin. As expected, H3K9me3-enriched domains correspond to the intensely DAPI-bright chromosomal core, consistent with models in which constitutive heterochromatin forms the structural scaffold of condensed chromosomes (37–39). At the same time, localization and distribution of constitutive heterochromatin coincides with the distribution of the major, high-copy, TmSat01 satDNA (**Figures 2A**, **3**). Within this framework, TmSat01 was robustly detected throughout both less and more condensed chromatin states (**Figure 2A**), imposing the idea that high repeat density can overcome steric and accessibility barriers characteristic for compacted chromatin. In contrast, detectability of low-copy satDNA varied with chromatin condensation level (**Figure 2B**), indicating that short, dispersed arrays are more prone to hybridization loss in condensed chromatin environments. Therefore, it is not surprising that satDNAs have traditionally been regarded as sequences inherently associated with heterochromatin, since the hybridization signals of major, easily detected, highly abundant satDNAs were often strong and colocalized with heterochromatic chromosomal regions.

It is now widely-acknowledged that satDNA display the diversity of their arrangements in a genome and scenarios underlying their origins [reviewed in (27, 40)]. SatDNA often localize outside classical heterochromatin domains, and in many insect species they associate with euchromatic or facultatively heterochromatic regions (e.g (41–46).). Similarly, mouse minor satDNAs, which are located at the centromere, are often detected as distinct individual spots on the periphery of the main heterochromatic block (constituted by the major satDNA), visible as punctate signals on the edge of the central mass (47).

Our results complement and extend these findings, demonstrating that low-copy satDNAs in *T. molitor* are physically extruded from the central chromosomal mass and occupy structurally distinct, loosely condensed domains. Here, recurrent pattern of signals located outside of the compact chromosomal mass was detected for several low-copy satDNA (**Figure 4**). These punctate signals appear to completely lie outside the DAPI-stained chromosomal core. However, inverted DAPI imaging reveals that they occupy distinct regions of very loosely condensed chromatin that forms extensions around the central heterochromatin. One possible explanation is that these satDNAs are located within chromatin loops extending from more compact regions, which could result in their apparent positioning toward the chromosomal periphery (**Supplementary Figure 1**). In this context, their distribution may arise as a consequence of spatial organization or some steric constraints, where low-abundance, short, and interspersed arrays are less likely to be incorporated into large heterochromatic blocks. It is also possible that their localization is influenced by the epigenetic state, as regions lacking heterochromatic marks such as H3K9me3 tend to remain less condensed and more spatially extended. It cannot be excluded that a synergistic action of mechanical loop extrusion and epigenetic state separation result in higher-order chromatin organization.

## 5 Conclusions

In conclusion, our results show that the cytogenetic detection and spatial distribution of satDNAs in *T. molitor* depend on multiple factors, including chromatin condensation, repeat abundance, the epigenetic state of chromatin, and overall chromosomal architecture. Highly abundant satDNAs constitute the H3K9me3-enriched constitutive heterochromatin forming the central chromosomal core and remain readily detectable even in highly condensed chromatin states. In contrast, low-copy satDNAs occupy regions of very loosely condensed chromatin outside of the chromosomal core, and are prone to signal loss. Overall, our study highlights the importance of considering chromatin context when interpreting satDNA localization. Together, these findings indicate that probe accessibility and hybridization efficiency are influenced not only by differential chromatin condensation, but also by the distinct epigenetic contexts, physical chromosomal architecture, and the positioning of heterochromatic and euchromatic domains, resulting in differences in satDNA detectability and spatial distribution between high- and low-copy satDNA families in *T. molitor*.

## Data Availability

The original contributions presented in the study are included in the article/**Supplementary Material**, further inquiries can be directed to the corresponding author/s.
