# Supplementary material for: Chromatin condensation dynamics during spermatogenesis and variation in detectability and spatial distribution of satellite DNAs outside constitutive heterochromatin in Tenebrio molitor
Source: Front Insect Sci. 2026 May 28;6:1858332. doi: 10.3389/finsc.2026.1858332 (PMC13253522; doi:10.3389/finsc.2026.1858332)
Supplement: Supplementary file 1 [file SupplementaryFile1.docx]

Supplementary Material

# Supplementary Table 1. Summary of cell types, division types and stages, and ploidy transitions during spermatogenesis.

| Cell type | Stages | Ploidy | DNA content |
| --- | --- | --- | --- |
| Spermatogonia | MitosisG1 → S → G2 → Prophase → Metaphase → Anaphase → Telophase → Cytokinesis | 2n | 2C → 4C (after S-phase) |
| Primary spermatocytes | Meiosis I (reductional)Premeiotic S-phase → Leptotene → Zygotene → Pachytene → Diplotene → Diakinesis → Metaphase I → Anaphase I → Telophase I | 2n | 4C |
| Secondary spermatocytes | Meiosis II (equational)Prophase II → Metaphase II → Anaphase II → Telophase II → Cytokinesis | n | 2C |
| Spermatids | Post-meiotic differentiationearly → late spermatid | n | 1C |
| Spermatozoa | Terminal differentiationto mature sperm | n | 1C |

# Supplementary Table 2. Primers and PCR conditions used for labelling FISH probes for satDNAs included in this study.

| Primer | Sequence (5’ – 3’) | Initial denaturation | Amplification (35 cycles) | Final extension |
| --- | --- | --- | --- | --- |
| TmSat01_F  TmSat01_R | GAATTCTGTAGTTCTTGCG  CAGGTTCCAAGACGC | 94°C 5' | 94°C 30''  48 °C 30'' 72°C 20'' | 72°C 5' |
| TmSat02_F  TmSat02_R | CGTAAGTTTCAATTATAACTCG  AGAATTTCCTGCCCAAAC |  |  |  |
| TmSat04_F  TmSat04_R | CTACGGCAATGAATTTTAC  CTTGCTTAATAACGGAGC |  |  |  |
| TmSat06_F  TmSat06_R | GATGATGTTGAACTAGACG  AGTAGAGCCAACGACC |  |  |  |
| TmSat08_F  TmSat08_R | ATGTTTGTGGGTGAAATC  AAACATCTTCCTATCGAC |  |  |  |


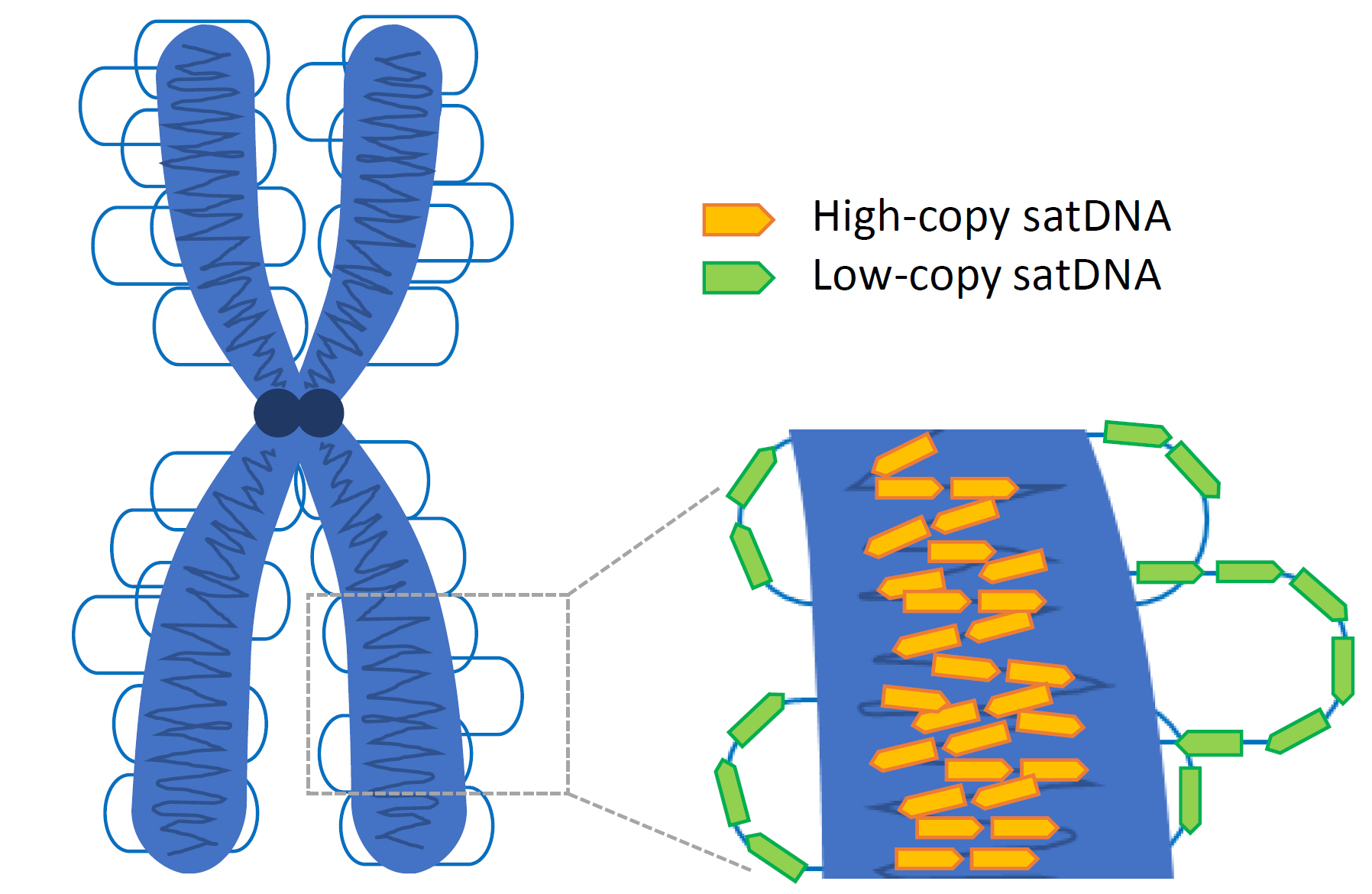


**Supplementary Figure 1.** Proposed model of low-copy satDNA positioning outside the central heterochromatic chromosomal core within loosely condensed chromatin loops extending from more compact chromosomal regions.
